# Innate immune gene expression in *Acropora palmata* is consistent despite variance in yearly disease events

**DOI:** 10.1371/journal.pone.0228514

**Published:** 2020-10-22

**Authors:** Benjamin D. Young, Xaymara M. Serrano, Stephanie M. Rosales, Margaret W. Miller, Dana Williams, Nikki Traylor-Knowles

**Affiliations:** 1 Department of Marine Biology and Ecology, Rosenstiel School of Marine and Atmospheric Science, University of Miami, Miami, FL, United States of America; 2 Atlantic Oceanographic and Meteorological Laboratory, National Oceanographic and Atmospheric Administration, Miami, Florida, United States of America; 3 Cooperative Institute for Marine and Atmospheric Studies, University of Miami, Miami, Florida, United States of America; 4 Southeast Fisheries Science Center, NOAA-National Marine Fisheries Service, Miami, FL, United States of America; 5 SECORE International, Miami, FL, United States of America; Helmholtz-Zentrum fur Ozeanforschung Kiel, GERMANY

## Abstract

Coral disease outbreaks are expected to increase in prevalence, frequency and severity due to climate change and other anthropogenic stressors. This is especially worrying for the Caribbean branching coral *Acropora palmata* which has already seen an 80% decrease in cover primarily due to disease. Despite the importance of this keystone species, there has yet to be a characterization of its transcriptomic response to disease exposure. In this study we provide the first transcriptomic analysis of 12 *A*. *palmata* genotypes and their symbiont Symbiodiniaceae exposed to disease in 2016 and 2017. Year was the primary driver of gene expression variance for *A*. *palmata* and the Symbiodiniaceae. We hypothesize that lower expression of ribosomal genes in the coral, and higher expression of transmembrane ion transport genes in the Symbiodiniaceae indicate that a compensation or dysbiosis may be occurring between host and symbiont. Disease response was the second driver of gene expression variance for *A*. *palmata* and included a core set of 422 genes that were significantly differentially expressed. Of these, 2 genes (a predicted cyclin-dependent kinase 11b and aspartate 1-decarboxylase) showed negative Log2 fold changes in corals showing transmission of disease, and positive Log2 fold changes in corals showing no transmission of disease, indicating that these may be important in disease resistance. Co-expression analysis identified two modules positively correlated to disease exposure, one enriched for lipid biosynthesis genes, and the other enriched in innate immune genes. The hub gene in the immune module was identified as D-amino acid oxidase, a gene implicated in phagocytosis and microbiome homeostasis. The role of D-amino acid oxidase in coral immunity has not been characterized but could be an important enzyme for responding to disease. Our results indicate that *A*. *palmata* mounts a core immune response to disease exposure despite differences in the disease type and virulence between 2016 and 2017. These identified genes may be important for future biomarker development in this Caribbean keystone species.

## Introduction

Since the 1980’s, the Caribbean has seen dramatic losses of hard coral cover [1,2]. This has been especially notable for *Acropora palmata* and *Acropora cervicornis* which have seen an 80% reduction throughout their geographic range [2]. This has resulted in them being classed as threatened (US Endangered Species Act; ESA), and critically endangered (IUCN). The primary cause of this decline is disease [2–4] and this is particularly worrying for these species as climate change and anthropogenic stressors are now being implicated in increasing disease prevalence, frequency, and severity [5–10]. While these two species are now being heavily used in restoration activities in the Caribbean, their disease susceptibility requires a more thorough understanding of the disease dynamics within the remnant populations.

Identifying the causative agents of coral disease has been a challenge. Similar disease phenotypes can be attributed to different causative agents [11] and these etiologies can shift over time and space [12]. This is in part due to corals being symbiotic organisms that host a diverse set of microbial partners [13,14] and disentangling the roles of beneficial versus pathogenic is complex [15]. A recent approach has been to use transcriptomics as a tool to understand the coral host’s response to disease [16–23]. This has allowed characterization of the hosts response to disease without knowing the exact causative agent(s), while also showing that the coral host’s innate immune system is activated and responding to a disease challenge [16–26]. By focusing on understanding the coral transcriptomic response, we can characterize the disease responses to a wide range of potential pathogens and identify core sets of genes that are activated regardless of pathogen stimulation. This will be particularly important in identifying signatures of disease resistance in coral species for restoration activities, while also providing potential diagnostic tools for coral health [27]. While different diseases may elicit unique responses in corals, we hypothesize that there will also be a core immune response of corals in response to infectious pathogens which can be measured using transcriptomics.

In 2016 and 2017, a disease grafting transmission study was performed in the Florida Keys using *A*. *palmata* [28]. This study found that in 2016 there was significant differences in disease transmission between genets, while in 2017 there was no observed differences in disease transmission between genets [28]. However, it was noted that there were differences in disease virulence between 2017 (average 80% transmission) and 2016 (average 30% transmission) which may have masked any genotypic responses. Histological analysis of corals identified white band disease (WBD) as the putative disease in 2016, while in 2017 rapid tissue loss (RTL) was identified as the putative disease [28]. 16s rRNA microbiome analysis was performed on the 2017 corals and identified *Sphingomonadaceae* as the putative causative pathogen [29]. The reasons for this observed increase in virulence are unknown, but it is hypothesized that it could be due to different diseases, baseline coral health, or an unknown environmental component [28].

To expand on these previous studies, we examined the transcriptomic response of *A*. *palmata* and their algal symbiont Symbiodiniaceae to identify the conserved and unique transcriptomic responses to disease in 2016 and 2017. We found that year was the overall strongest driver of gene expression for both *A*. *palmata* and the symbiont Symbiodiniaceae reflecting the observed different diseases previously identified in 2016 and 2017 [28]. We identified genes that we hypothesize may indicate dysbiosis or compensation between the coral host and Symbiodiniaceae. The response to disease was the second strongest driver of gene expression in *A*. *palmata*, but not in Symbiodiniaceae, with clear expression profiles between exposed samples showing no disease signs (No Transmission) and disease signs (Transmission). We believe this indicates that *A*. *palmata* initiates a core immune response to disease exposure regardless of the diseases identified in 2016 and 2017. There were sets of unique and common significantly differentially expressed genes shared between No Transmission and Transmission samples. These genes included innate immune and cell adhesion genes. Within the common genes, only two (Predicted cyclin-dependent kinase 11B and aspartate 1-decarboxylase) showed different expression profiles (positive in No Transmission and negative in Transmission). We hypothesize that these could be important for disease resistance and should be studied further. Co-expression analysis identified two statistically significant modules ('Brown' and 'Skyblue') which positively correlated to disease exposure. The 'Brown' module, which was enriched for innate immune processes, identified D-amino acid oxidase as the hub gene. D-amino acid oxidase is an important immune modulator previously unstudied in corals and may be an important target for future research. Overall, this study has provided the first characterization of the transcriptomic response to disease in *A*. *palmata* and has identified new gene targets that may be useful as coral health biomarkers.

## Methods

### Disease grafting experiment and genotype selection

For transcriptomic analysis, 12 *A*. *palmata* genets with previously published transmission information were analyzed [28]. In 2016 and 2017, disease grafting experiments were performed at the Coral Restoration Foundation (CRF; Tavernier Offshore Nursery) using 12 genets of *A*. *palmata* that are actively used for outplanting projects [28]. All Field experiments and sample collections were performed under Florida Keys National Marine Sanctuary permit #FKNMS-2016-024-A1.

**Fig 1:**
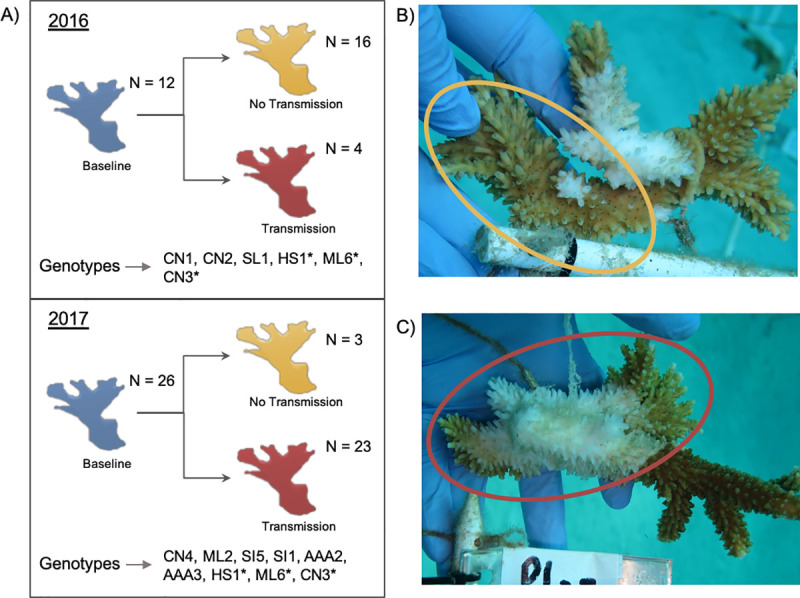
Experimental summary for transcriptomic analysis. A) A general overview of the field experiment conducted in 2016 and 2017. Samples were taken before disease grafting (blue = Baseline) and after 7-days of disease grafting, with samples classified as showing no signs of disease transmission (yellow = No Transmission) or signs of disease transmission (red = Transmission). Genotypes sequenced are listed below. Genets with a * were tested in 2016 and 2017. B) The yellow circle indicates the apparently visually healthy *A*. *palmata* fragment grafted to the diseased *A*. *cervicornis* fragment after exposure for 7-days resulting in it being classed as No Transmission. C) The red circle indicates *A*. *palmata* fragment showing disease signs grafted to the diseased *A*. *cervicornis* fragment after 7 days resulting in it being classed as Transmission.

Coral fragments for this study were placed on an isolated coral tree structure, away from the main propagation nursery [28]. Fragments of *A*. *palmata* were grafted to diseased fragments of *A*. *cervicornis* over 7-days to identify disease transmission rates between the different genets [28]. Seven days has previously been identified to show transmission for disease in *Acropora* species [21] and was thus used in the disease transmission experiment [28]. *A*. *cervicornis* disease inoculants were chosen according to gross visual signs [28]. A set of controls (healthy *A*. *cervicornis* grafted to *A*. *palmata* fragments) were also run at the same time as the disease grafts. These controls showed no signs of active disease, indicating that disease transmission resulted from the disease grafts attached [28]. A ~1cm^2^ piece of tissue was clipped from the base of each *A*. *palmata* fragment. These were taken before disease grafting (Baseline) and after 7-days exposure to either disease or control. After 7-days of exposure, fragment disease outcomes were scored as follows: No Transmission (no visible disease signs, Fig 1B) or Transmission (visible disease signs, Fig 1C). Samples were then either flash frozen in liquid nitrogen (2016), or placed in RNAlater (2017), and stored at -80°C. In total for transcriptomic analysis, there were 32 samples in 2016 and 52 in 2017 (Table 1 and Fig 1A). Of the 12 total genets, three (HS1, ML6 and CN3) were assayed in both 2016 and 2017 to examine any impacts of each year on gene expression and ensure it was not due to genotypic variation (Fig 1A and Table 1).

**Table 1:** Breakdown of genets and fragments sequenced for gene expression analysis.

CRF Genet Names	Miller *et al* (2019) Genet Names [28]	Year	Baseline	No Transmission	Transmission	Total
CN1	P2	2016	1	3	0	4
CN2	P4	2016	2	3	1	6
SL1	P5	2016	2	3	0	5
HS1*	P1	2016	3	3	0	6
ML6*	P3	2016	2	1	3	6
CN3*	P6	2016	2	3	0	5
HS1*	P1	2017	3	1	2	6
ML6*	P3	2017	3	0	2	5
CN3*	P6	2017	3	0	3	6
CN4	P7	2017	3	1	2	6
ML2	P8	2017	3	0	3	6
SI5	P9	2017	3	0	3	6
SI1	P10	2017	3	0	3	6
AAA3	P11	2017	3	0	3	6
AAA2	P12	2017	2	1	2	5

**Fig 2:**
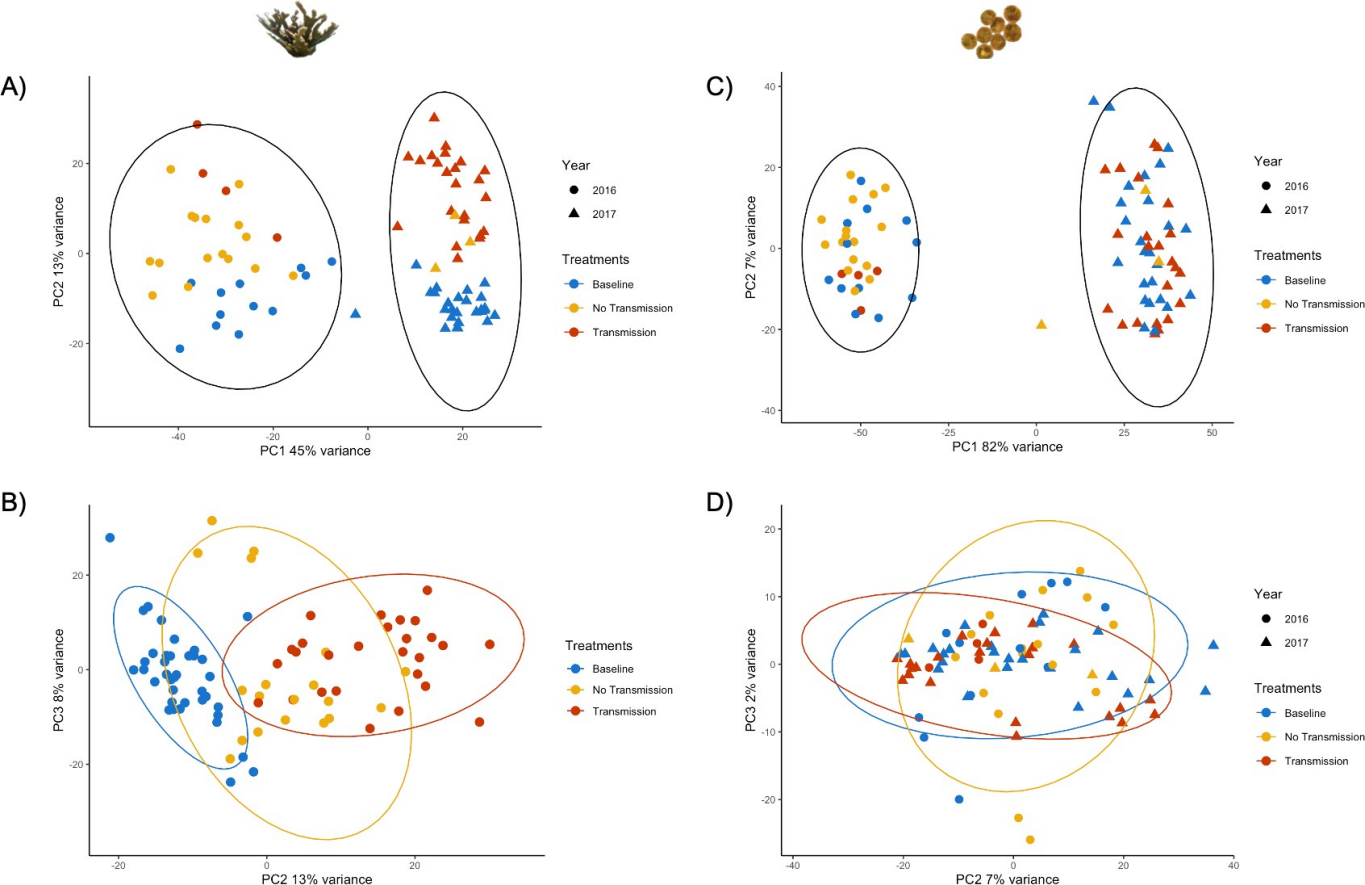
*A*. *palmata* and Symbiodiniaceae samples cluster by year, while disease response is only identified in the coral. A) Principal Component (PC) 1 and PC2 of *A*. *palmata* counts, using a variance stabilizing transformation (VST), identifies the difference between years as the primary driver of sample variance. B) PC2 and PC3 of *A*. *palmata* counts, using a VST, is driven by disease response. C) PC1 and PC2 of Symbiodiniaceae counts, using a VST, identifies year as the primary driver of sample variance. D) PC2 and PC3 of Symbiodiniaceae counts, using a VST, shows no effect of disease outcome. For A) and C), black ellipses represent a 95% confidence interval in 2016 and 2017. For B) and D), the colored ellipses represent 95% confidence intervals for Baseline (blue), No Transmission (yellow) and Transmission (red).

**Fig 3:**
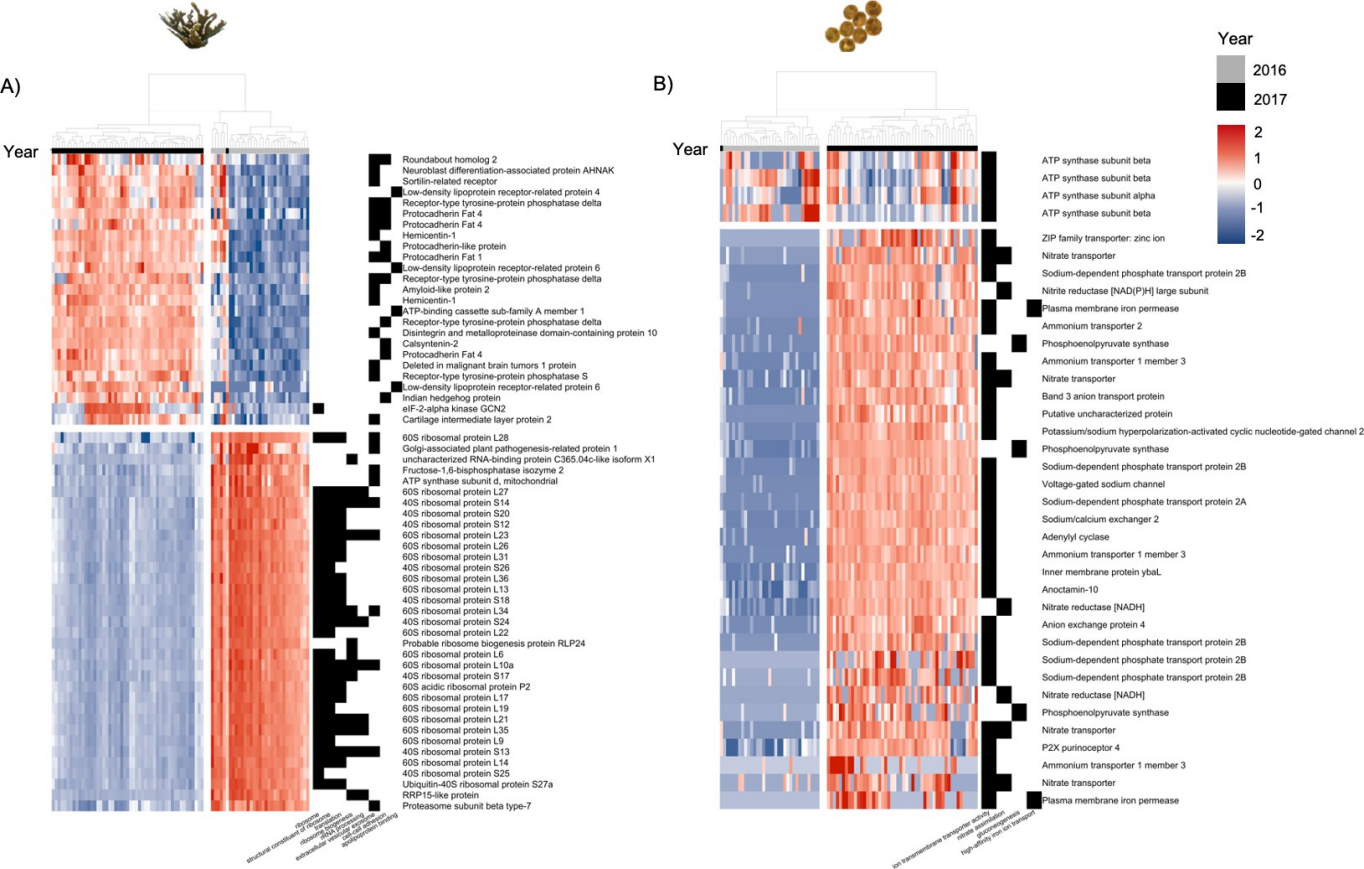
Genes driving the difference between 2016 and 2017 responses in the coral host and Symbiodiniaceae. A) Coral host genes linked to significantly enriched gene ontology (GO) terms, identified from principal component (PC) 1 loadings. Genes are linked to translation and ribosomal formation processes. Hierarchical clustering of the samples (heatmap columns) shows grouping between the samples from 2016 (grey) and 2017 (black), with 2016 genes having higher normalized expression and 2017 having lower normalized expression. B) Symbiodiniaceae genes linked to significantly enriched GO terms identified from PC1 loadings. Genes are linked to transmembrane ion transport processes. Hierarchical clustering of the samples (heatmap columns) shows grouping between the samples from 2016 (grey) and 2017 (black). For A) and B), grey = 2016 samples, black = 2017 samples. Left heatmap shows higher (red) to low (blue) gene counts using a variance stabilizing transformation. Right heatmap is presence (black) and absence (white) of genes to GO terms. Column dendrogram shows hierarchical clustering of samples. Rows (genes) also arranged using hierarchical clustering with dendrogram omitted.

**Fig 4:**
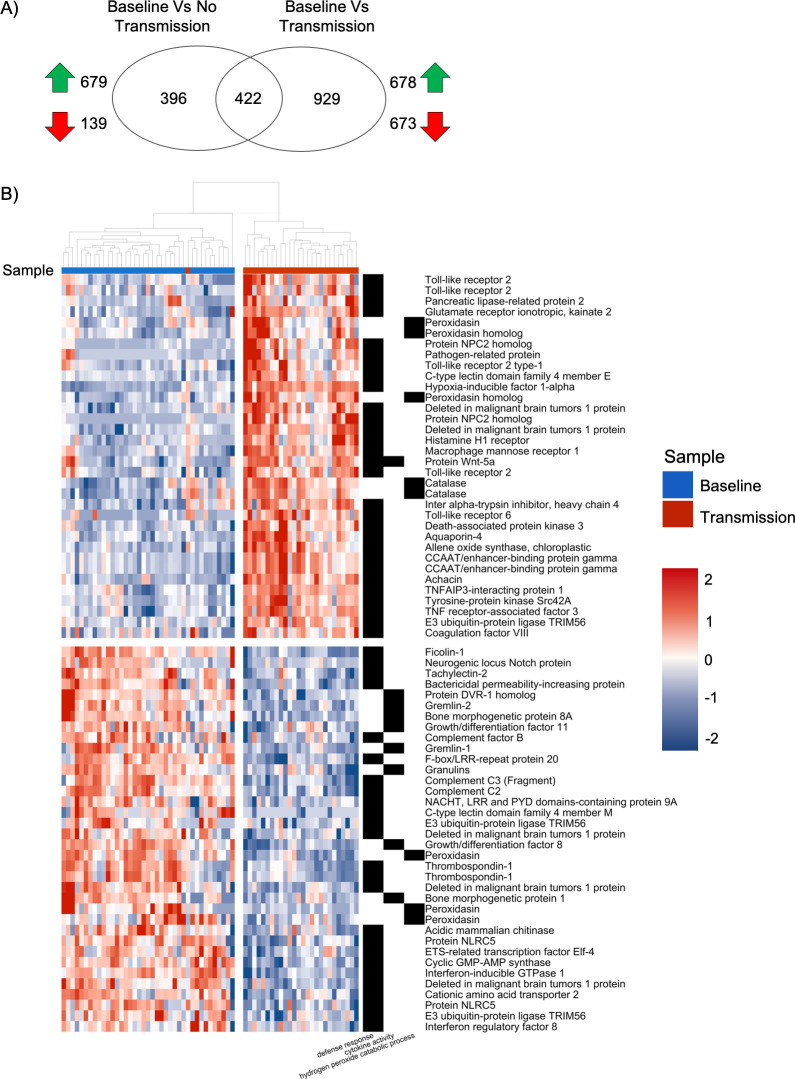
Unique and common genes between differential expression contrasts and significant innate immune genes in diseased corals. A) Venn diagram of the unique (left and right) and shared (intersect) differentially expressed genes from the two contrast arguments run in DeSeq2. The green arrows show significantly upregulated and the red arrows show significantly downregulated genes for each contrast. B) Heatmaps showing genes linked to significantly enriched innate immune gene ontology (GO) terms identified from the Baseline vs. Transmission DeSeq2 contrast. Samples included are Baseline (blue) and Transmission (red). Left heatmap fill shows higher (red) to low (blue) gene counts using a variance stabilizing transformation. Right heat map identifies genes present (black) or absent (white) from significantly enriched GO terms linked to innate immune response. Column dendrogram shows hierarchical clustering of samples. Rows (genes) also arranged using hierarchical clustering with dendrogram omitted.

Shading for Baseline (blue), No Transmission (yellow) and Transmission (red) is the same for Figs 1–4. Middle section including HS1*, ML6* and CN3* are the genets present in 2016 and 2017. To allow comparison between this and the previous study [28], please see genet names listed. In this paper we use the CRF genet designations.

### cDNA library preparation and sequencing

In total 88 samples, consisting of Baseline, No Transmission and Transmission samples, were selected for transcriptomic analysis. All samples were processed for total RNA using the Qiagen RNeasy Minikit with the recommended 15-minute DNase digestion. Total RNA quality and quantity were assessed using a Nanodrop and Qubit fluorometer. Total RNA was then converted to complementary DNA (cDNA) libraries using Illumina TruSeq RNA Library poly A-tail selection prep kit following the manufacturer protocol. During cDNA library preparation, Illumina adaptors were randomly assigned to samples to reduce bias between sequencing lanes. cDNA libraries were quantified using a Qubit fluorometer and sent to the Utah Huntsman Cancer Institute High Throughput Genomics Shared Resource Center. cDNA quality control was performed using High Sensitivity D100 Screentape. A total of 84 samples passed quality control and were sequenced for 50 base pair single-end reads on 4-lanes using an Illumina HiSeq 2500 (Fig 1A and Table 1).

### Bioinformatic analysis

Sequenced libraries were processed following standard practices for RNA-seq analysis [30]. All program parameters and scripts are available at https://github.com/benyoung93/apal_disease_transcritpomics. Read quality was assessed using FastQC [31] and low-quality reads were trimmed using Trimmomatic [32]. Trimmed reads were then aligned to the A. palmata genome assembled from reads previously reported [33] using STAR [34] with the provided GFF file used for gene annotation and function [35]. Because A. palmata shows stable symbioses within the genus Symbiodinium (formerly Clade A [36]) over time and space [37], reads that did not align to the A. palmata genome were aligned to a Symbiodinium annotated transcriptome [38]. A. palmata and Symbiodiniaceae aligned reads where then quantified using Salmon [39] before being read into R (v3.6.1) and RStudio (v1.2.1335) using tximport [40]. While pre-filtering is not needed due to DeSeq2 performing independent filtering of low count and low power genes [41], we incorporated a pre-filtering step to reduce memory requirements and increase speed. Pre-filtering for A. palmata (less than 1 count in greater than 15 samples), and for Symbiodiniaceae (less than 1 count in greater than 20 samples) was done using the counts per million (CPM) function in EdgeR [42]. Pre-filtered counts were then used for differential gene expression analysis and co-expression analysis.

### Coral and Symbiodiniaceae principal component analysis

Sample counts were transformed using the variance stabilizing transformation (VST) function in DeSeq2 [41] and used as input for principal component analysis (PCA). A modified ‘PlotPCA’ function from DeSeq2 [41] was used to identify sample distribution for *A*. *palmata* and Symbiodiniaceae over multiple principal components (PCs) and plotted using ggplot2 [43]. To identify genes driving sample grouping in the PCA, loadings were extracted for PCs, and any genes with a +/- 2 standard deviation (SD) were retained for Gene Ontology (GO) analysis. We used a +/- 2 SD to have a non-biased cut-off that was the same for each set of genes identified from *A*. *palmata* and Symbiodiniaceae.

### Coral host differential expression between Baseline and disease outcomes

DeSeq2 [41] was used to analyze differential gene expression for the *A*. *palmata* quantified transcripts. The model ~Year + Group was used to account for batch effects caused by different preservation methods used between the different years, while ‘Group’ encompassed Baseline and disease outcomes: No Transmission and Transmission. This removed variance from the years and allowed significantly differentially expressed genes only due to disease outcome to be analyzed. Using this model, subsequent pairwise comparisons were performed using the ‘contrast’ function in DeSeq2 [41] between experimental outcomes; Baseline vs. No Transmission, and Baseline vs. Transmission. Genes that were significantly differentially expressed (DEGs) had a false discovery rate (FDR) adjusted p-value <0.01, and a Log2 fold change (L2FC) >1 or <-1. These sets of DEG were used in GO analysis.

Results from the two contrasts (Baseline vs. No Transmission, and Baseline vs. Transmission) were then analyzed to identify any shared genes present. The L2FC for each contrast was compared to identify any differences in expression directionality due to disease outcome, and the full set of common genes were used in GO analysis.

### Weighted gene co-expression network analysis

To identify groups of co-expressed transcripts that correlated to Baseline and disease outcomes, a weighted gene coexpression network analysis (WGCNA; [44]) was used. Due to disease outcome being identified on PC axis 2 (Fig 2B), the variance due to the year was removed using ‘removeBatchEffect’ in the program Limma [45]. Input data was therefore the CPM filtered batch removed counts with a VST for all 84 samples. Initial clustering using the Ward method in WGCNA [44] indicated there were no outlier samples and allowed retention of all 84 samples for co-expression analysis. A single signed network was built with manual network constructions (Key parameters: soft power = 12, minimum module size = 40, deep split = 2, merged cut height = 0.40, minimum verbose = 3, cutHeight = 0.997). The eigengene values of each module were correlated to treatment: Baseline, No Transmission, Transmission. To identify the highest connected gene within each module (hub gene), the WGCNA [43] command ‘chooseTopHubInEachModule’ was used. All significant modules (alpha < 0.05) were then used in subsequent GO analysis.

### Gene ontology analysis

To identify significant enrichment of Gene Ontology (GO) terms (biological process, cellular component, and molecular function) Cytoscape v3.7.2 [46], with the add-on application BiNGO [47], was used. The hypergeometric test was utilized for GO enrichment and p-values were corrected with a Benjamini & Hochberg false discovery rate (FDR) correction (alpha set at < 0.01). For *A*. *palmata*, the full mRNA transcriptome from the *A*. *palmata* genome [33,35] was used as the background set of genes for the enrichment tests. For Symbiodiniaceae, the full transcriptome [38] was used as the background set of genes for the enrichment tests. GO visualization was then done in Cytoscape v3.7.2 [46] allowing identification of significantly enriched relationships between parent and child terms. Genes in significantly enriched GO terms of interest were then visualized in RStudio using the VST counts and Complex Heatmap [48].

## Results

### Sequencing depth, read alignment, assignment metrics

A total of 84 samples were successfully sequenced on 4-lanes of an Illumina HiSeq 2500 with an average single-end read depth of 10,808,777. All raw reads are available on NCBI (SRA PRJNA529682). From quality filtered sequences, 74.64% of single end reads mapped to the *A*. *palmata* genome [33,35] using STAR [34]. Quantification, using Salmon [39], resulted in 35,079 genes having at least one count across all samples, with subsequent CPM filtering (less than 1 count in >15 samples) reducing this to 18,913 genes for downstream analysis. Of reads not aligning to the *A*. *palmata* genome [33,35], an average of 21.54% aligned to the *Symbiodinium* (Clade A) reference transcriptome [38] using STAR [34]. Quantification using Salmon [39] yielded counts for 72,152 transcripts, with 28,035 of these retained for downstream analysis after CPM filtering (less than 1 count in greater than 20 samples).

### Year was the greatest determination of gene expression for *A*. *palmata* and Symbiodiniaceae with ribosomal and ion transport genes driving sample clustering

PCA showed *A*. *palmata* samples clustered by year on PC 1 (PC1 = 45%; Fig 2A), followed by disease outcome on PC 2 (PC2 = 13%; Fig 2B). Symbiodiniaceae samples also clustered by year on PC1 (PC1 = 82%; Fig 2C) while PC 2 showed no correlations to disease exposure or genet (Fig 2D).

Analysis of the genes driving PC1 variance for *A*. *palmata* identified 86 significantly enriched GO processes; 48 Biological Process, 6 Molecular Function, and 32 Cellular Components. Within Biological Process and Cellular Component, genes associated with ribosomal structure and function, as well as ribosomal RNA processing were significantly enriched. Three GO terms were also linked to immune processes; cell-cell adhesion, extracellular vesicular exosome, and apolipoprotein binding. Visualization of the VST counts for the genes within these GO terms identified 4 heatmap clusters (Fig 3A). All genes linked to ribosomal processes showed lower normalized counts in 2017 than 2016, while GO terms with potential immune genes and functions showed higher normalized counts in 2017 than in 2016 (Fig 3A). PC1 loadings and full GO results for *A*. *palmata* are available in S1 Table.

For Symbiodiniaceae, there were 120 significantly enriched GO processes; 48 Biological Process, 6 Molecular Function, and 32 Cellular Components. In all three GO components, significantly enriched terms identified 2 main gene processes. Genes implicated in the transport of ions between cells and cellular components showed higher expression in 2017 than in 2016 (Fig 3B). This included plasma membrane iron permease, nitrate and nitrite transporters, sodium transporters, zinc transporters, and ammonium transporters. Genes linked to photosynthesis, namely photosystems I and II in the light dependent reaction, also showed significant GO enrichment. The genes within these photosynthesis terms did not exhibit higher or lower expression compared between year, but instead showed a range of expression across the samples for each year (S1 Fig). PC 1 loadings and full GO results for Symbiodiniaceae are available in S2 Table.

### Significant differential gene expression was identified between different disease outcomes in *A*. *palmata*

Differential gene expression analysis was only done for *A*. *palmata* due to there being no disease response identified in the Symbiodiniaceae through PCA (Fig 2B and 2D). For Baseline vs. No Transmission, there were 139 transcripts significantly downregulated, and 679 transcripts significantly upregulated, while Baseline vs. Transmission had 673 transcripts significantly downregulated and 678 transcripts significantly upregulated (adjusted p-value <0.01, L2FC >1 or <-1; Fig 4A). Full lists of significant DEG for each contrast are available in S3 and S4 Tables respectively. Between each contrast, there were 422 shared differentially expressed transcripts (Fig 4A). Of these, only 2 showed opposite LFC directionalities; a ‘predicted cyclin-dependent kinase 11B-like partial’ (Baseline vs. No Transmission L2FC = 2.57, Baseline vs. Transmission L2FC = -1.98), and an Aspartate 1-decarboxylase (Baseline vs. No Transmission L2FC = 1.53, Baseline vs. Transmission L2FC = -2.18). A full list of shared genes with L2FC is available in S5 Table.

### Contrast between Baseline and no Transmission

The significant DEGs for the contrast between Baseline vs. No Transmission showed significant enrichment of 18 GO terms: 4 Biological Process, 7 Molecular Functions, and 7 Cellular Components (S3 Table). Biological Processes identified enrichment of the GO term cell surface receptor linked signaling pathways which included a number of putative immune function genes such as: tumor necrosis factor (TNF) receptor-associated factor 3, WNT proteins, protein kinase C epsilon type, MAPK-activating death protein, and genes involved in recognition such as apolipophorin, C-type lectins and a number of adhesion G-coupled protein receptors (GPCRs) (S2 Fig). Additionally, significant enrichment of the GO term cell adhesion was identified. This included a number of mucin proteins, a brevican core protein, collagen alpha chains, tenascin-X and R, Sushi von Willebrand factors, protocadherin-like proteins, and proto-oncogene tyrosine-protein kinase receptors (S2 Fig). Genes with putative immune functions within the cell adhesion GO term were also identified including TNF alpha induced protein 3 and protein kinase proteins (S2 Fig). Cellular component enriched GO terms identified genes associated with the extracellular matrix and the plasma membrane, and identified additional putative immune function genes: toll like receptor (TLR) 6, scavenger receptors, and macrophage mannose receptor (S2 Fig). All significant GO terms and associated genes are available in S3 Table.

### Contrast between Baseline and Transmission

The significant DEGs for the contrast between Baseline vs. Transmission showed significant enrichment of 46 Biological processes, 14 Cellular Component, and 35 Molecular Function (S4 Table). GO terms linked to defense response, bioluminescence, and cytokine activity contained innate immune genes including four genes similar to toll-like receptor (TLR) 2, and two genes similar to TLR 6 complexes (Fig 4B). There were also lectin pathway recognition genes: c-type lectin domain family 4 member E and M, ficolin-1. Other receptors which have been implicated in innate immunity were also identified: F-box/LRR-repeat protein 20, histamine H1 receptor, macrophage mannose receptor 1, two NOD-like receptor proteins, and a neurogenic locus notch protein (Fig 4B). Innate immune genes involved in signaling including TLR signaling pathway components were also identified, such as: deleted in malignant brain tumor 1, CCAAT/enhancer-binding protein gamma, gremlin 1 and 2, NACHT LRR and PYD domain contain proteins 12 and 9A, TNF receptor-associated factor 3, TNFAIP3-interacting protein 1, and E3 ubiquitin-protein ligase TRIM56 (Fig 4B). There were also genes important in lectin signaling: complement C2 and C3. Finally, there were antimicrobial peptides (AMPS) such as achacin, bactericidal permeability-increasing protein, and a pathogen related protein (Fig 4B). Lastly, three transcription factors were also identified: CCAAT/enhancer-binding protein gamma, Interferon-inducible GTPase 1 and interferon regulatory factor 8 (Fig 4B). All significant GO terms and associated genes are available in S4 Table.

### Co-expression analysis identifies positively correlated modules of immune genes and lipid biosynthetic processes to disease exposure

**Fig 5:**
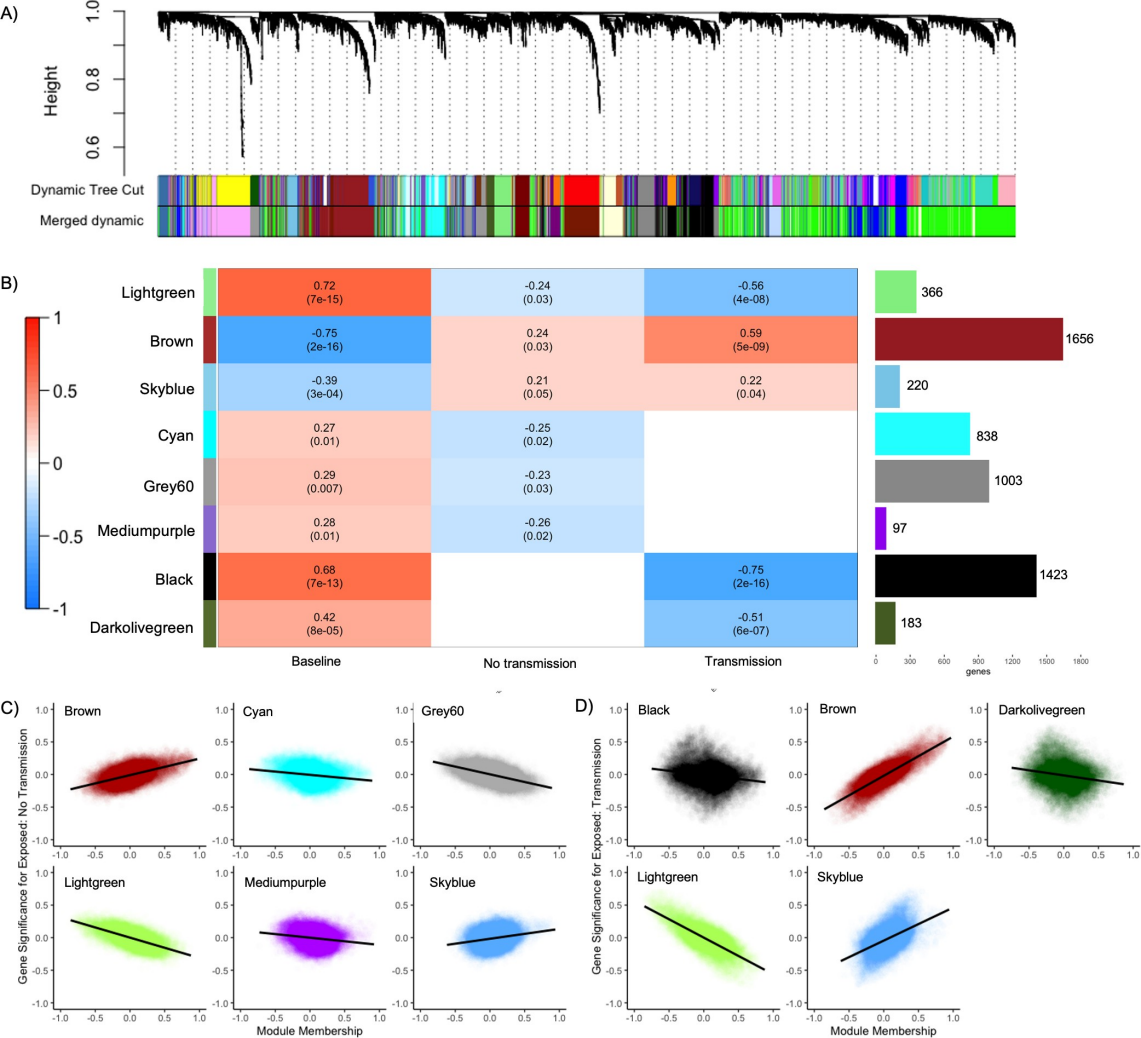
Co-expression analysis identifies 19 gene modules, with eight significantly correlated to Baseline, Transmission and No Transmission. A) Dynamic tree height showing merging of modules with similar expression patterns. Merging resulted in the 43 original modules (Dynamic Tree Cut) being merged into 19 modules (Merged Dynamic). B) Co-expression heatmap showing eight modules that are significantly correlated among Baseline, No Transmission, and Transmission ('Lightgreen', 'Brown', 'Skyblue'); Baseline and No Transmission ('Cyan', 'Grey60', and 'Mediumpurple'); and Baseline and Transmission ('Black' and 'Darkolivegreen'). Heatmap fill shows positive (red) to negative correlation (blue). The top number in each cell shows the correlation strength and the bottom number shows module significance to Baseline, No Transmission, and Transmission. Bar graph to the right shows the number of genes within each module. C) The six modules that are significantly correlated between Baseline and No Transmission showing the module membership and gene significance. D) The five modules that are significant between Baseline and Transmission showing the module membership and gene significance. For C and D: Y-axis shows gene significance that is the absolute value of the correlation between the gene and disease outcome. X-axis shows the module membership which is the correlation of the module eigengene and the gene expression profile.

After merging of similar modules (Fig 5A), we identified 19 co-expressed modules (S3 Fig) that contained 76 to 2027 genes. Of these 19 modules, 8 showed significant correlations to Baseline, No Transmission, and Transmission (Fig 5B). Gene lists for significant modules are provided in S6 Table.

Of the 19 modules, ‘Lightgreen’ (366 genes, hub gene = Interferon Regulatory Factor 2), ‘Brown’ (1656 genes, hub gene = D-amino-acid oxidase), and ‘Skyblue’ (220 genes, hub gene = PREDICTED: uncharacterized protein LOC107335116) were all significantly correlated (p≤0.05) across Baseline, No Transmission, and Transmission (Fig 5B–5D). These modules were significantly enriched (FDR, p<0.01) for multiple GO Biological Processes, Cellular Components, and Molecular Functions with 58 terms for the ‘Brown’ module, 1 for ‘Skyblue’, and 0 for ‘Lightgreen’. The ‘Brown’ module had a negative correlation for Baseline (R^2^ = -0.75) but was positively correlated for No Transmission (R^2^ = 0.24) and Transmission (R^2^ = 0.59) (Fig 5B). The ‘Brown’ module was significantly enriched for terms in immune processes such as TLR-6 signaling pathway, MyD88-dependent signaling pathway, positive regulation of cytokine biosynthetic processes, detection and response to diacylated bacterial lipopeptide, podosome, phagocytic and endocytic vesicle membranes, and lipopeptide binding. The ‘Skyblue’ module was significantly enriched for only lipid biosynthetic processes and also showed positive correlations with No Transmission (R2 = 0.21) and Transmission (R^2^ = 0.22) (Fig 5B). All genes for significant modules are available in S6 Table, genes linked to significant GO terms for significant modules are available in S7 Table.

Three modules were significantly correlated to Baseline and No Transmission; ‘Cyan’ (838 genes, hub gene = F-box/LRR-repeat protein 7), ‘Grey60’ (1003 genes, hub gene = Isopentenyl-diphosphate Delta-isomerase 1), and ‘Mediumpurple’ (97 genes, hub gene = pyridoxine-5`-phosphate oxidase) at p≤0.05 (Fig 5B and 5C). The ‘Cyan’ module was significantly enriched (FDR, p<0.01) for 40 GO Biological Processes that included genes involved in cell adhesion, immune responses (complement activation, leukocyte mediated immunity, regulation of coagulation), and metabolic/catabolic processes but showed negative correlations with disease outcomes (Fig 5C). ‘Mediumpurple’ was enriched for GO terms involved in respiration (electron transport chain, oxidative phosphorylation, ATP synthesis) as well as biosynthetic processes and the positive regulation of necrotic cell death. ‘Grey60’ was enriched for three GO terms, including cellular metabolic processes, nitrogen compound metabolic processes and cellular nitrogen compound metabolic processes. All genes for each module are available in S6 Table and genes linked to significant GO terms are available in S7 Table.

Two modules were significantly correlated to Baseline and Transmission: ‘Black’ (1423 genes, hub gene = Ufm1-specific protease 2) and ‘Darkolivegreen’ (183 genes, hub gene = S-adenosylmethionine decarboxylase proenzyme) at p≤0.05 (Fig 5B and 5D). The ‘Black’ module was significantly enriched with one GO term, metabolic processes, while ‘Darkolivegreen’ module was not significantly enriched for any GO terms. All genes for each module are available in S6 Table, and genes linked to significant GO terms are available in S7 Table.

## Discussion

### Gene expression differences between years shows evidence of a compensation or dysbiosis between the coral host and Symbiodiniaceae

Previously, differences in disease type (WBD 2016 and RTL in 2017) and virulence between the grafting experiments run in 2016 and 2017 were documented [28]. Through our gene expression analysis, we also identified a clear difference in gene expression of the samples between 2016 and 2017 in both the coral host (Fig 2A), and the Symbiodiniaceae (Fig 2C) [28]. Without physiological or abiotic measurements, a correlative mechanism cannot be determined, but we hypothesize that the split between 2016 and 2017 may be due to an unknown abiotic stress that has influenced the baseline health of the coral [49,50]. This hypothesis was formed due to a common disease response in the coral host (identified on PC 2) despite the different disease types previously reported [28] (Fig 2B). This hypothesis, however, should be thoroughly tested in the future by measuring abiotic and physiological parameters to ascertain the baseline health of *A*. *palmata*.

The overall response of the Symbiodiniaceae to disease is still not well understood. Previous studies have found confounding results for photochemical efficiency, indicating that the role of the Symbiodiniaceae varies [11,51]. However, one previous study has shown that Symbiodiniaceae gene expression is affected by pathogen exposure, indicating that it is possible for Symbiodiniaceae to be affected by coral pathogens [52]. Here, we identified differences between 2016 and 2017 (Fig 2C), but no response to disease (Fig 2D). Our gene expression data identified higher expression profiles for ion transmembrane transporter activity genes in 2017 than 2016 (Fig 3B). We hypothesize that this is indicative of either a compensation response or potential dysbiosis [14,53]. The increased gene expression of transporter proteins in 2017 may indicate that Symbiodiniaceae is trying to maintain their nutrient needs that are not being met by the coral host. Conversely, increases of these transporter proteins may be indicative of a dysbiosis between the Symbiodiniaceae and the coral host, causing the Symbiodiniaceae to move towards a parasitic state [53,54]. In the future, incorporating abiotic measurements, photosynthetic efficiency metrics, and symbiont density counts will help in our understanding of this potentially important interaction.

### Despite disease differences in 2016 and 2017, there is a core immune response between Baseline, No Transmission, and Transmission corals

Our study characterizes a core immune response in *A*. *palmata* despite differences in expected disease types and differences in disease transmission rates between the two years (Fig 2B) [28]. PC2 identified distinct groupings of Baseline and Transmission samples, while No Transmission samples were intermediate between Baseline and Transmission (Fig 2B). No Transmission corals had no visual signs of disease, like Baseline corals, and maintained similar expression profiles with the exception of putative pathogen resistance pathways such as cell adhesion and immune signaling pathways (S3 Table). We hypothesize, based on this finding, that No Transmission corals behave similarly to Baseline corals. Conversely, Transmission corals showed active disease signs and a larger change in gene expression, presumably based on an active disease response mounted by the coral immune system.

### Enrichment of cell adhesion genes was found in No Transmission corals

For No Transmission, the DeSeq2 contrast identified 679 significantly upregulated and 139 significantly downregulated genes. These genes were enriched for the biological process GO terms: “Cell Adhesion” and “Cell surface receptor linked signaling pathways”, with these genes enriched within the extracellular matrix and plasma membrane (S3 Table, S2 Fig). In previous coral disease studies, significant GO enrichment of cell adhesion was present in corals that were showing signs of disease pathology and hypothesized to be due to the importance of apoptotic processes and phagocytosis of melanized particles and pathogens [18,19]. Our findings show that cell adhesion may not only be important for corals exhibiting disease signs, but also important in corals not exhibiting visual signs of disease. In the future, characterization of these cell adhesion genes should be investigated as they may provide sets of genes to use as diagnostic tools for resistant corals.

### Corals with visual disease transmission activate an innate immune response

Transmission samples had enrichment for GO terms involved in innate immune response including “Defense Response”, “Cytokine Activity”, and “Bioluminescence” (Fig 4B, S4 Table). Our results are similar to previous transcriptomic studies, where innate immunity genes were upregulated in response to disease transmission [16–23]. We identified significantly upregulated TLR 2 and TLR 6 genes which are important innate immune pattern recognition receptors (PRR) that identify gram-negative bacteria and fungi respectively [55,56]. These receptors are important for initiating the Nuclear Factor Kappa Beta (NF-kB) transcription factor that causes production of cytokines and AMPS [57–61]. While other components of the NF-kB pathway were not significantly differentially expressed in this study, they are present in the *A*. *palmata* genome [33,35] and have been functionally characterized in the coral *Orbicella faveolata* [62].

Our differential expression results also identified AMP transcripts including a bactericidal permeability-increasing protein (BPIP) and achacin. BPIP kills gram negative bacteria by targeting the lipopolysaccharide outer cell membrane [63–67]. Achacin, an AMP present in African Giant Slug mucus, has potent gram-positive and gram-negative bacteria killing properties [68–70]. To our knowledge, these AMPs have not been characterized in any other coral disease studies but could be important targets for coral disease defense. These AMPs are present in the *A*. *cervicornis* genome [33,35] but, on re-annotation of a similar experiment run in *A*. *cervicornis* [20] with the *A*. *cervicornis* genome [33,35], these genes were not significantly differentially expressed at alpha 0.01 or 0.05 (re-annotation pipeline and subsequent results available at https://github.com/benyoung93/apal_disease_transcritpomics). With these AMPs present in the genome, but not differentially expressed in *A*. *cervicornis*, we hypothesize that they are evolutionarily conserved but may play a more important role in *A*. *palmata* disease response than in *A*. *cervicornis*.

Significant differential expression was also identified for five lectins, including c-type lectin domain family 4 member E and M, ficolin-1, and tachylectin-2, and macrophage mannose receptor 1. These lectins are important in symbioses recognition and maintenance [71–73] while also being implicated in pathogen recognition and subsequent complement pathway activation [19,22,71,74]. Our findings support previous studies that lectins play a complex role in both symbiosis and pathogen recognition in corals, however, the specific mechanisms and pathways these lectins initiate are still not well understood. A number of genes were also identified as potential macrophage immune factors. Cationic amino acid transporter has been identified to have a role in macrophage immunity [75], while tyrosine-protein kinase Src42a has been shown to promote macrophages to sites of wounding [76]. Previous studies in sponges have identified potential macrophage expressed protein activity [77,78] with high amino acid conservation with human macrophage expressed proteins. The presence of a similar gene in corals indicates that this immune process may be conserved not only in sponges and humans, but also in corals. While invertebrates do not have an adaptive immune system, the presence of this gene may be indicative of phagocytosis managing pathogen infection in *A*. *palmata*.

### Only two genes show different Log2 fold changes between Baseline and disease exposure contrasts

Of the 422 genes shared between the DeSeq2 contrasts for Baseline vs. Transmission and Baseline vs. No Transmission, only two showed differing L2FC: PREDICTED cyclin-dependent kinase 11B-like partial, and Aspartate 1-decarboxylase. Both showed positive L2FC in No Transmission (2.57 and 1.53 respectively), and negative L2FC in Transmission (-1.98 and -2.18 respectively).

Aspartate 1-decarboxylase synthesizes β-alanine is needed for the biosynthesis of pantothenate [79]. Pantothenate is used in the synthesis of coenzyme A, which has key roles in enzyme activation and deactivation through acylation and acetylation, as well as signal transduction. Pantothenate deficiency has been implicated in disorders of the nervous, gastrointestinal, and immune systems [80]. In corals, downregulation of genes involved in pantothenate metabolic process have been observed in corals 10 hours after heat stress [81] and have been hypothesized to increase host susceptibility to pathogens due to downregulation of innate immune responses through pantothenate deficiency [81,82]. In our findings, corals that had increased L2FC of Aspartate 1-decarboxylase showed no signs of disease. This indicates that pantothenate biosynthesis could be an important process in eliciting a successful immune response to disease exposure in *A*. *palmata*. We hypothesis that this gene, and pantothenate metabolic processes in general, are important for disease resistance in *A*. *palmata* and future investigation into the mechanisms and relationship with the innate immune system should be investigated.

Cyclin-dependant kinase 11B is expressed in proliferating cells [83] and is an important signal for modulating gene transcription and cell division [84]. Cyclin-dependent kinase 11B has been implicated in hormone receptor signalling and autophagy [85–87]. Recently, cyclin-dependent kinases have been shown to be important in innate immune responses [88], having roles in type I interferon (IFN) activity [89] and tumor necrosis factor-induced NF-kB activity [90]. We hypothesize that this gene may play a currently undescribed role in *A*. *palmata*'s innate immune response, with further research looking to characterise its activity in type I IFN and TNF-induced NF-kB activity [88–90].

### Lipid biosynthesis may play a key role in the activation and maintenance of an immune response in *A*. *palmata*

The ‘Skyblue’ coexpression module showed a positive correlation with disease outcome (Fig 5C and 5D) and significant enrichment of the GO term “lipid biosynthetic processes”. We hypothesize that this, coupled with the differential gene expression between Baseline vs. Transmission, indicates that *A*. *palmata* was mounting an energetically expensive immune response to the disease challenge. Stored energy, in the form of lipids, can be metabolized and assist in promoting a stronger inflammatory response for fighting off pathogens [91]. This idea has been proposed in other transcriptomic studies on coral disease [23], indicating that this could be integral to multiple coral species’ disease responses. In the future, linking specific *A*. *palmata* genotype lipid production and storage with disease susceptibility may be an important metric for understanding their capacity of resistance and recovery, as seen during coral bleaching [92–94].

### ‘Brown’ module is rich in innate immune genes and the hub gene, D-amino acid oxidase, is a critical immune factor involved in *A*. *palmata* disease response

The 'Brown' coexpression module showed increasing positive correlations with disease outcomes (Fig 5C and 5D), including significant enrichment of innate immunity genes (S6 Table) and GO terms (S7 Table). This module shows great overlap with other coral disease transcriptomic studies and cnidarian immune responses, including TLR signaling and TNF [17,18,20,21,23], NOD-like receptors (NLRs) [16], lectin pathways [19], and AMPs [16,17]. Within the 'Brown' module, D-amino acid oxidase (DAO) was identified as the hub gene. DAO is a peroxisomal enzyme important for mammalian mucosal microbiome homeostasis and leukocyte phagocytosis [95–98]. During bacterial phagocytosis, free floating D-amino acid (DAA) released by bacteria is sensed by G-protein coupled receptors in phagocytes [95]. DAO is then released into the phagosome, catalyzing the deamination of DAA which release hydrogen peroxide and kills the bacteria [96,98]. Its presence as a hub gene correlated with disease response could indicate that it is a critical coral immune factor that has previously been overlooked. In a similar experiment in *A*. *cervicornis*, DAO is differentially expressed between healthy and diseased corals [20]. Interestingly, their study showed a large positive L2FC of 7.83 which is similar to what we identified in the contrast between Baseline and Transmission (L2FC = 6.22). In other coral species, DAO was not identified as significantly differentially expressed [16,17,19,23] indicating it may be a critical immune factor in the genus *Acropora*. There are seven GPCRs also present in the 'Brown' module (S6 Table), indicating that a similar interaction of DAO and GPCRs may be occurring in *A*. *palmata* as what has been observed in other organisms [95,98]. We hypothesize that DAO, through GPCR recognition, may be an important immune response in *Acropora* and should be investigated further.

## Conclusions and future directions

Historically, coral disease research has focused on identifying the causative pathogens of coral disease, with only a handful of studies fulfilling Koch’s postulates (see reviews [11,15]). This approach has proven difficult due to pathologies of diseases being attributed to different causative pathogens [11,99] while disease etiologies from the same pathogen being misidentified [12]. Here we present further evidence that using transcriptomics to identify the host’s response to disease exposure can still be valuable despite not knowing putative causative agent(s). We identified year as the primary cause of gene expression variance, which mirrored the identified increased virulence seen in the field [28]. We believe this is showing a compensatory or dysbiosis between the host and Symbiodiniaceae, but this merits future research with abiotic and physiological data collection in tandem with transcriptomics. Despite the observed differences in disease type and virulence between 2016 and 2017 [28], we have identified a core immune response for *A*. *palmata* that is consistent between the two years. This includes a wide repertoire of immune genes that have been identified in other coral disease transcriptomic studies [16–23], as well as new novel genes that have not been previously described in coral disease literature. We also show two genes, cyclin-dependent kinase 11B and aspartate 1-decarboxylase, which could be important genes for disease resilience due to their opposite expression profiles in Transmission and No Transmission corals. Lastly, we identified that sets of genes involved in lipid biosynthesis and immune responses are crucial for the disease response through co-expression analysis. This also identified DAO as a hub gene with important implications in coral immune response.

Overall, this work has expanded our understanding of the of innate immune response of corals to disease. It has also provided the first transcriptomic disease analysis of the critically endangered *A*. *palmata* and can help inform future restoration efforts through continued disease-based experiments. This work has important implications for restoration practitioners for informing outplant survivability through the development of novel diagnostic markers.

## Supporting information

S1 Fig Heatmap of Symbiodiniaceae genes associated with photosynthetic GO terms. For left heatmap grey = 2016 samples, black = 2017 samples. Fill shows higher (red) to low (blue) gene counts using a variance stabilizing transformation. Column dendrogram shows hierarchical clustering of samples. Rows (genes) also arranged using hierarchical clustering with dendrogram omitted. Right heatmap is presence (black) and absence (white) of genes to GO terms.
(TIF)

S2 Fig Heatmap of significant GO terms in corals showing no disease signs. Heatmaps showing genes linked to significantly enriched interesting gene ontology (GO) terms identified from the Baseline vs. No Transmission DeSeq2 contrast. Samples included are Baseline (blue) and No Transmission (yellow). Left heatmap fill shows higher (red) to low (blue) gene counts using a variance stabilizing transformation. Right heat map identifies genes present (black) or absent (white) from significantly enriched GO terms. Column dendrogram shows hierarchical clustering of samples. Rows (genes) also arranged using hierarchical clustering with dendrogram omitted.
(PNG)

S3 Fig Co-expression heatmap for the 19 modules identified from WGCNA analysis. Heatmap fill shows positive (red) to negative correlation (blue). Rows are identified modules from coexpression analysis. Columns are treatments (Baseline, No Transmission, and Transmission). The top number in each cell shows the correlation strength, and the bottom number shows module significance in relation to experimental treatment (Baseline, No Transmission, and Transmission).
(TIF)

S4 Fig (TIF)

S1 Table *A*. *palmata* Principal Component 1 gene loadings and GO list with associated genes. (XLSX)

S2 Table Symbiodiniaceae Principal Component 1 gene loadings and GO list with associated genes. (XLSX)

S3 Table Baseline vs. No Transmission DeSeq2 results and significant GO terms with associated genes. (XLSX)

S4 Table Baseline vs. Transmission DeSeq2 results and significant GO terms with associated genes. (XLSX)

S5 Table Shared genes between DeSeq2 contrasts. (XLSX)

S6 Table Gene lists for significant modules from WGCNA analysis. (XLSX)

S7 Table GO terms and associated genes for significant WGCNA modules. (XLSX)
