# Supplementary figures and images for: Innate immune gene expression in Acropora palmata is consistent despite variance in yearly disease events
Source: PLoS One. 2020 Oct 22;15(10):e0228514. doi: 10.1371/journal.pone.0228514 (PMC7580945; doi:10.1371/journal.pone.0228514)

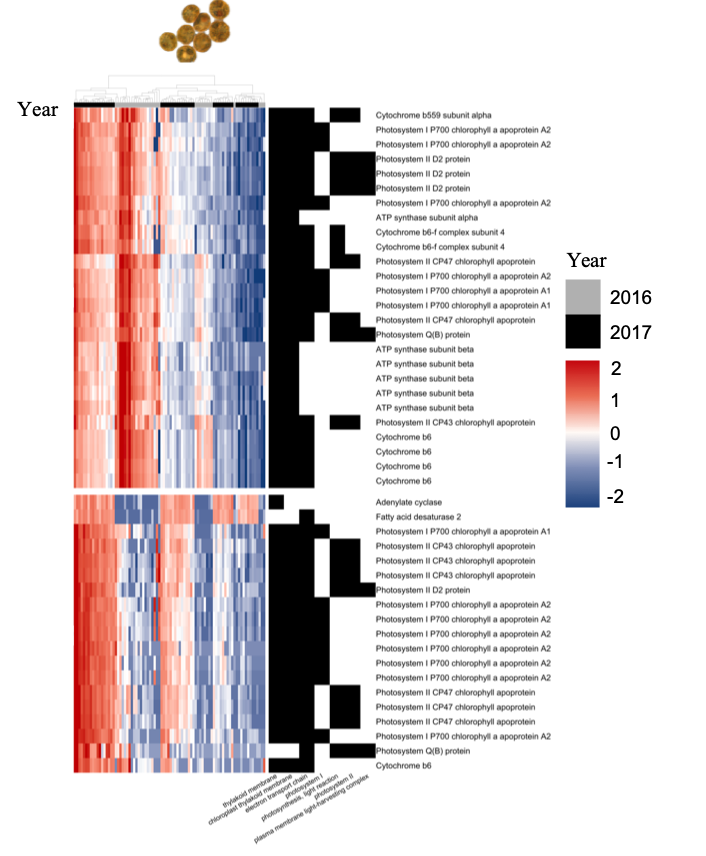

Supplement: S1 Fig — For left heatmap grey = 2016 samples, black = 2017 samples. Fill shows higher (red) to low (blue) gene counts using a variance stabilizing transformation. Column dendrogram shows hierarchical clustering of samples. Rows (genes) also arranged using hierarchical clustering with dendrogram omitted. Right heatmap is presence (black) and absence (white) of genes to GO terms. (TIF) [file pone.0228514.s001.tif]

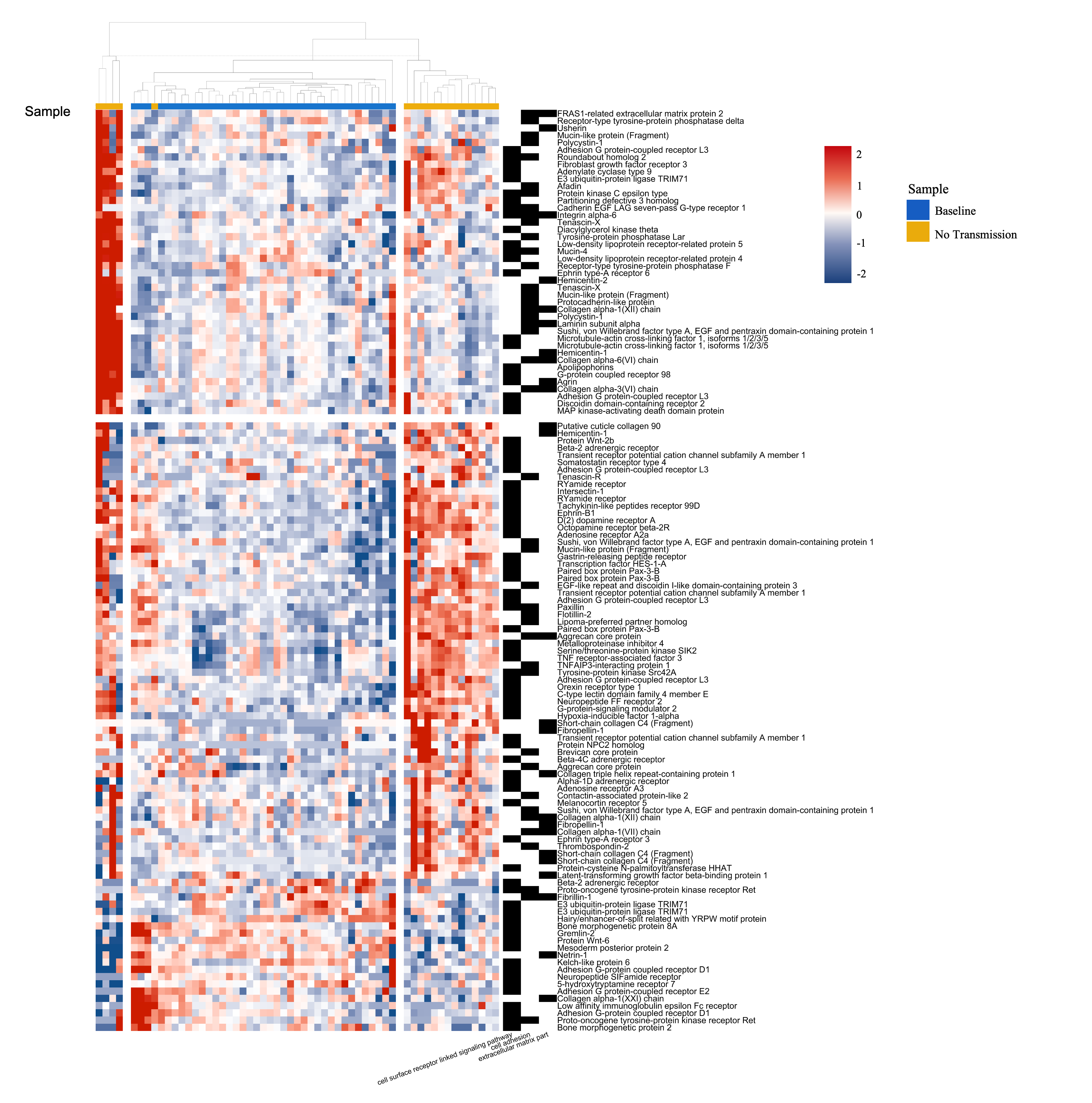

Supplement: S2 Fig — Heatmaps showing genes linked to significantly enriched interesting gene ontology (GO) terms identified from the Baseline vs. No Transmission DeSeq2 contrast. Samples included are Baseline (blue) and No Transmission (yellow). Left heatmap fill shows higher (red) to low (blue) gene counts using a variance stabilizing transformation. Right heat map identifies genes present (black) or absent (white) from significantly enriched GO terms. Column dendrogram shows hierarchical clustering of samples. Rows (genes) also arranged using hierarchical clustering with dendrogram omitted. (PNG) [file pone.0228514.s002.png]

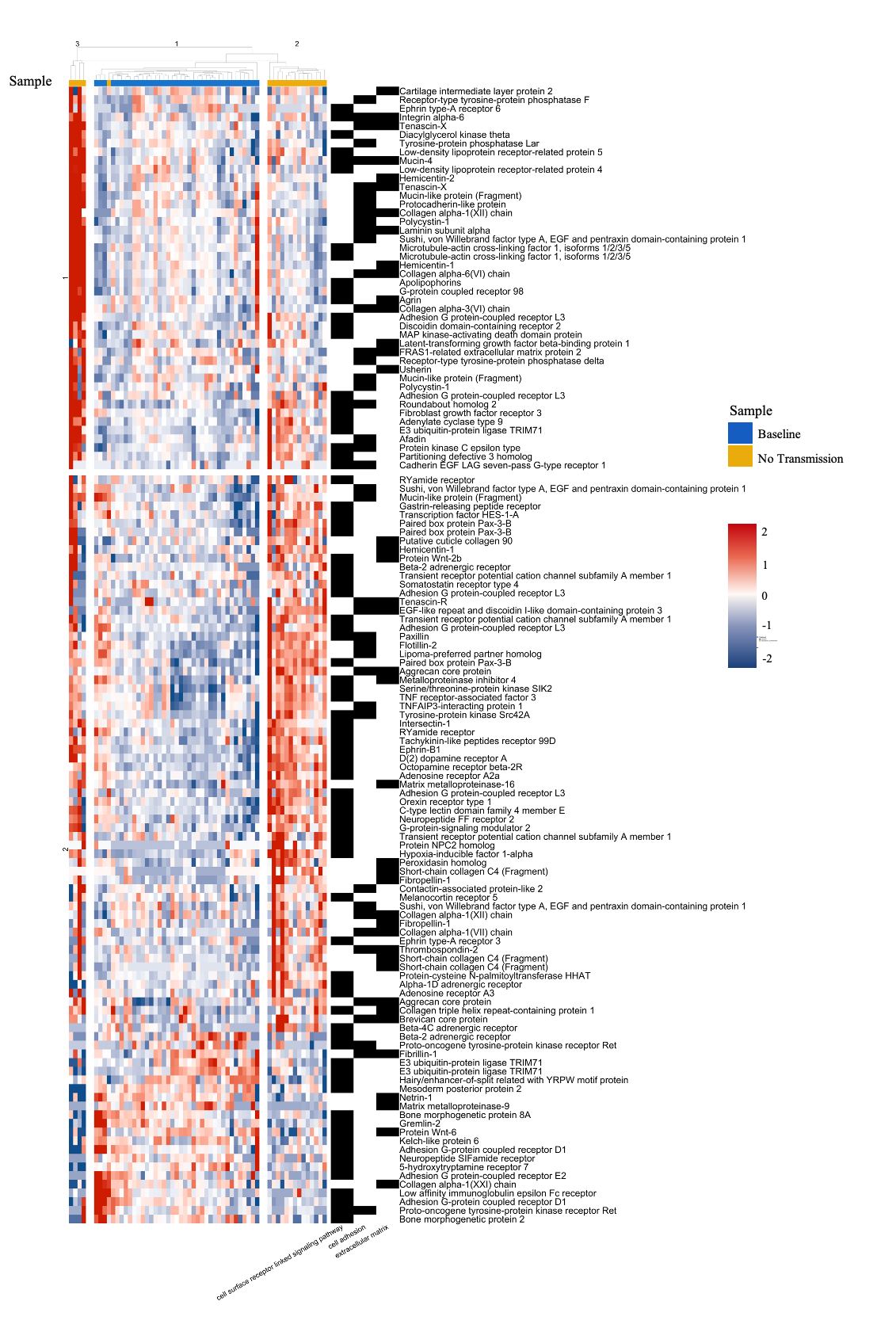

Supplement: S3 Fig — Heatmap fill shows positive (red) to negative correlation (blue). Rows are identified modules from coexpression analysis. Columns are treatments (Baseline, No Transmission, and Transmission). The top number in each cell shows the correlation strength, and the bottom number shows module significance in relation to experimental treatment (Baseline, No Transmission, and Transmission). (TIF) [file pone.0228514.s003.tif]

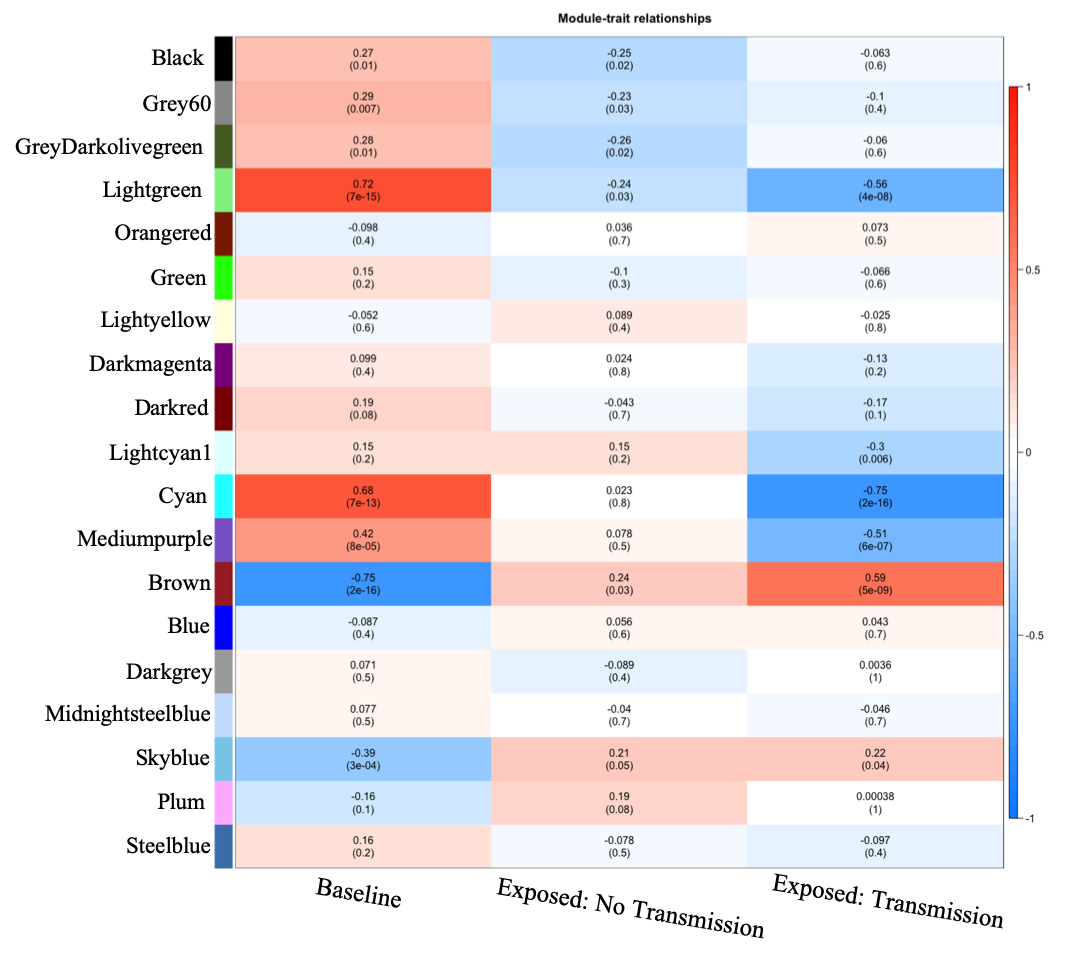

Supplement: S4 Fig — (TIF) [file pone.0228514.s004.tif]
